# *Mapping cardiac drug transport: In vitro* assessment of cardiac P-glycoprotein function with [^18^F]MC225 by using µ-engineered heart tissues

**DOI:** 10.1093/ehjopen/oeaf150

**Published:** 2025-11-25

**Authors:** Wanling Liu, Maureen Dannenberg, Pascalle Mossel, Zeyu Cai, Inês Farinha Antunes, Jurgen Sijbesma, Chantal Kwizera, Lu Cao, Andries van der Meer, Robert Passier, Riemer H J A Slart, Verena Schwach, Gert Luurtsema

**Affiliations:** Department of Nuclear Medicine and Molecular Imaging, University of Groningen, University Medical Center Groningen, Hanzeplein 1, Groningen 9713 GZ, The Netherlands; Applied Stem Cell Technologies, Department of Bioengineering Technologies, TechMed Centre, University of Twente, Drienerlolaan 5, Enschede 7500 AE, The Netherlands; Department of Nuclear Medicine and Molecular Imaging, University of Groningen, University Medical Center Groningen, Hanzeplein 1, Groningen 9713 GZ, The Netherlands; Department of Nuclear Medicine and Molecular Imaging, University of Groningen, University Medical Center Groningen, Hanzeplein 1, Groningen 9713 GZ, The Netherlands; Department of Nuclear Medicine and Molecular Imaging, University of Groningen, University Medical Center Groningen, Hanzeplein 1, Groningen 9713 GZ, The Netherlands; Department of Nuclear Medicine and Molecular Imaging, University of Groningen, University Medical Center Groningen, Hanzeplein 1, Groningen 9713 GZ, The Netherlands; Department of Nuclear Medicine and Molecular Imaging, University of Groningen, University Medical Center Groningen, Hanzeplein 1, Groningen 9713 GZ, The Netherlands; Leiden Institute of Advanced Computer Science (LIACS), Universiteit Leiden, Niels Bohrweg 1, Leiden 2333 CA, The Netherlands; Applied Stem Cell Technologies, Department of Bioengineering Technologies, TechMed Centre, University of Twente, Drienerlolaan 5, Enschede 7500 AE, The Netherlands; Applied Stem Cell Technologies, Department of Bioengineering Technologies, TechMed Centre, University of Twente, Drienerlolaan 5, Enschede 7500 AE, The Netherlands; Department of Anatomy and Embryology, Leiden University Medical Centre, PO Box 9600, Leiden 2300 RC, The Netherlands; Department of Nuclear Medicine and Molecular Imaging, University of Groningen, University Medical Center Groningen, Hanzeplein 1, Groningen 9713 GZ, The Netherlands; Department of Biomedical Photonic Imaging, University of Twente, Drienerlolaan 5, Enschede 7500 AE, The Netherlands; Applied Stem Cell Technologies, Department of Bioengineering Technologies, TechMed Centre, University of Twente, Drienerlolaan 5, Enschede 7500 AE, The Netherlands; Department of Nuclear Medicine and Molecular Imaging, University of Groningen, University Medical Center Groningen, Hanzeplein 1, Groningen 9713 GZ, The Netherlands

**Keywords:** P-glycoprotein, P-gp, Engineered heart tissues, Heart on a chip, Positron emission tomography (PET), [^18^F]MC225

## Abstract

**Aims:**

P-glycoprotein (P-gp), an efflux transporter with diverse compound effects, is a vital part of cardiac function. To determine if the selective substrate tracer [^18^F]MC225 also functions in cardiac P-gp, micro-engineered heart tissues (µ-EHTs) utilizing human induced pluripotent stem cell (hiPSC)-derived cardiomyocytes were used. This model offers advantages in potentially reducing animal experiments and allowing direct evaluation on human cells. However, its adoption in nuclear medicine remains very limited. This study aims to evaluate [^18^F]MC225 as a measurement method for cardiac P-gp function using a heart-on-chip model.

**Methods and results:**

µ-EHTs were treated with the P-gp inhibitor Tariquidar (200 nM for 30 min) or the P-gp inducer Doxorubicin (1 µM for 24 h) and incubated with [^18^F]MC225 (1 MBq/mL for 30 min). First, we identified and confirmed the expression of P-gp in the µ-EHTs using immunofluorescent staining, which showed an increase of P-gp expression after Doxorubicin treatment. According to γ-counter measurements, Tariquidar-treated tissues exhibited a higher uptake (117.5 ± 33.67%, *n* = 24) (*P* = 0.035) than the control, compared to Doxorubicin-treated tissues which exhibited a lower uptake (63.97 ± 21.89%, *n* = 20) (*P* < 0.001) compared to its controls. Autoradiography visualized radioactive distribution in each µ-EHT and confirmed the γ-counter measurements.

**Conclusion:**

[^18^F]MC225 effectively evaluates and measures cardiac P-gp function in µ-EHTs on the heart-on-chip platform. This research sets the stage for future studies using P-gp function to evaluate the efficacy and safety of novel cardiovascular drugs using µ-EHTs.

## Graphical Abstract

**Graphical Abstract:**
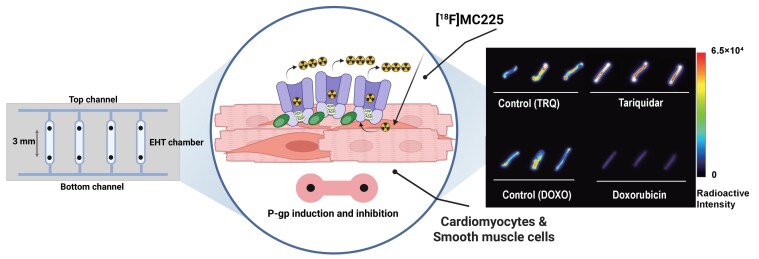

## Introduction

**Figure 1:**
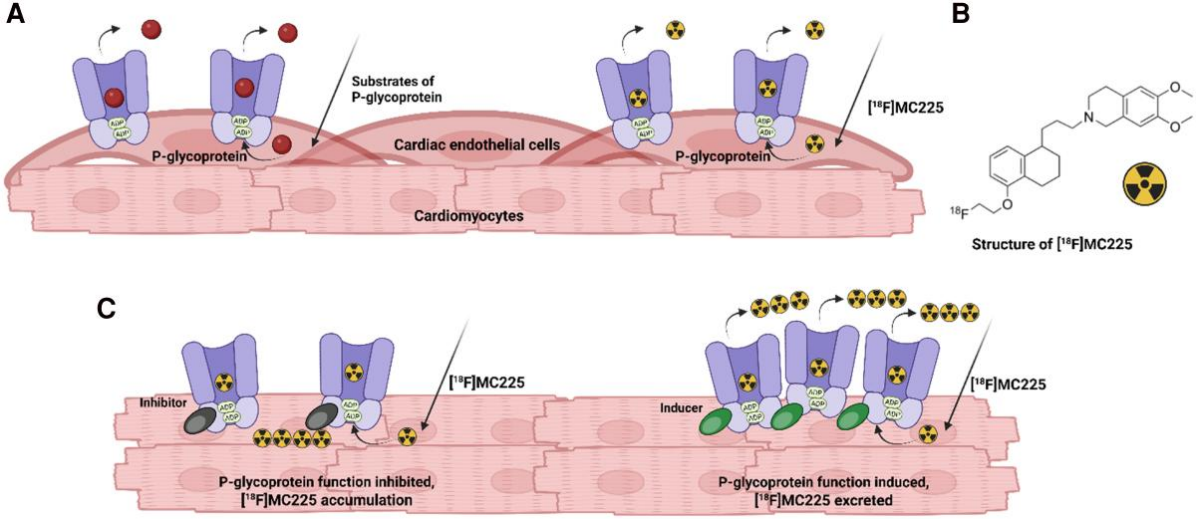
Function of P-gp transporter [^18^F]MC225 *in vivo vs. in vitro*. (*A*) The P-gp efflux transporters that are present in the cardiac endothelial cells *in vivo* pump its substrates out of the cells. [^18^F]MC225 acts as a substrate for the P-gp efflux transporter. (*B*) The chemical structure of [^18^F]MC225. (*C*) When P-gp transporters are inhibited *in vitro*, the efflux of [^18^F]MC225 is reduced, causing it to accumulate inside the cells. When P-gp transporters are induced *in vitro*, e.g. by cardiotoxic Doxorubicin, more [^18^F]MC225 is pumped out of the cells.

Transporters, crucial in regulating intracellular and extracellular drug concentrations, influence the efficacy of pharmacological treatments. P-glycoprotein (P-gp) is herein a key efflux transporter in humans, facilitating the transport of diverse, structurally unrelated compounds (P-gp substrates) out of cells.^1^ P-gp is expressed in several organs, including the brain, gut, and heart. In the heart, it plays an essential role in protection by managing the absorption and elimination of potentially toxic substances and drugs, as shown in *Figure 1A*. Notably, many cardiovascular medications including antiarrhythmics, antihypertensives, and anticoagulants, are P-gp substrates and their bioavailability is therefore affected by changes in P-gp function.^2,3^ This highlights P-gp's crucial function in regulating medication availability in the heart and makes it an interesting target for *in vivo* measurements.

Several factors, including genetic variations, physiological changes, diseases, and medications, can influence P-gp expression in the human heart. Meissner *et al.* observed a decrease in P-gp expression in patients with dilated cardiomyopathy, based on their study of 15 human cardiac samples.^4^ Moreover, pharmaceuticals can either inhibit or induce P-gp activity, leading to potential drug-drug interactions, overdoses, and adverse effects. Doxorubicin (DOXO) is a well-known cardiotoxic anthracycline that is widely applied for the treatment of cancer in millions of patients every year. Unfortunately, Doxorubicin treatment is often limited by its major toxicity in healthy organs, including the heart. Therefore, Doxorubicin is often used in *in vitro* models to assess the function of cardiomyocytes and drug-induced cardiotoxicity.^5^ Budde *et al.*^6^ demonstrated that Doxorubicin-treated mice exhibit cardiac upregulation of P-gp at both expression and functional levels. Therefore, the upregulation of P-gp following Doxorubicin treatment would be of interest to evaluate in current 3D *in vitro* models to track P-gp function, similar to *in vivo* conditions. Alterations in P-gp function can impact the therapeutic outcomes of cardiovascular treatments; enhanced P-gp activity can reduce drug efficacy, while diminished function can increase the risk of cardiotoxicity due to higher myocardial drug accumulation. Therefore, accurately assessing cardiac P-gp function is of vital importance.

Studying cardiac P-gp functionality is crucial for a deeper understanding of pathophysiological mechanisms of the (diseased) heart and how it affects drug bioavailability. The tracer [^18^F]MC225 has already proven its efficacy for measuring the function of P-gp at the human blood-brain barrier. However, it was never used for measuring cardiac P-gp function.^7,8^ [^18^F]MC225 is promising as PET tracer for assessing P-gp function in BBB and potentially for broader clinical applications, including the evaluation of cardiac P-gp function (*Figure 1C*).^9^

Animal models have been widely used in assessing the specificity and sensitivity of novel PET tracers *in vivo*. However, many studies find that species differences in P-gp function should not be disregarded among humans, rats, and mice.^10,11^ Recently, various 3D organ-on-chip models have been developed to recapitulate tissue-level functionality and communication between diverse types of cells and extracellular matrices.^12^ For example, engineered heart tissues (EHTs) can be used for cardiotoxicity drug screening.^5,13^ In our previously established μ-EHTs model, human induced pluripotent stem cells (hiPSC)-derived cardiomyocytes (CMs), fibroblasts (FBs), endothelial cells (ECs), and smooth muscle cells (SMCs) were incorporated. In this study, we aim to utilize μ-EHTs to replicate the physiological conditions of the human heart, enabling the simulation of human cardiac P-gp activity and functionality *in vitro*. This is the first evaluation of the new radioactive tracer using organ-on-chip models, potentially reducing the reliance on animal models.

In this study, the objective was to validate the 3D-µ-EHTs for measuring cardiac P-gp function using [^18^F]MC225. The project aims to study the feasibility of a heart-on-chip model and [^18^F]MC225 to measure cardiac P-gp function. For validation, this is done under 3 conditions—control, inhibition with tariquidar, and induced using Doxorubicin. It may enhance the evaluation of novel drugs and optimize future therapeutic strategies in cardiovascular medicine through the heart-on-chip platform.

## Material and methods

### HPSC culture

The experiments were performed using the human embryonic stem cell (hESC) line with dual cardiac reporter NKX2.5^EGFP/+^-ACTN2-^mRubyII/+14^ and the human induced pluripotent stem cell (hiPSC) lines LU54 (LUMC0054iCTRL#2)^15^ and WTC-11 (WT GM25256*G0002).^16^ HiPSCs and hESCs were maintained as undifferentiated colonies in Essential 8 medium (Thermo Fisher Scientific, A1517001) on vitronectin (Thermo Fisher Scientific, A31804)-coated 6-well plates (Greiner Bio-One, 657 160). HiPSCs were passaged with 0.5% Revitacell (Thermo Fisher Scientific, A2644501), and hESCs without Revitacell.

### HiPSC differentiation to cardiomyocytes

Cardiomyocyte differentiation of WTC-11 was induced in monolayer culture as described before in,^17^ with cells seeded at 2.5 × 10^4^ cells/cm^2^ on Matrigel (Corning, 354 230)-coated 6-well plates in Essential 8 medium one day before starting the differentiation. On differentiation day 0, cardiac mesoderm was induced by the medium with BPEL supplemented with 20–30 ng/mL BMP4 (R&D Systems), 20–30 ng/mL Activin A (Miltenyi Biotec) and 1.5–2.25 μM CHIR99021 (Axon Medchem). The medium was refreshed on differentiation day 3 with BPEL supplemented with 5 μM XAV939 (R&D Systems), and subsequently on differentiation days 6 and 10 with BPEL medium only. On differentiation days 13 and 15, cells were refreshed with CM medium with triiodothyronine hormone (100 nM, Sigma-Aldrich, T6397), insulin-like growth factor 1 (100 ng/mL, Bio-Connect, C-60839), and dexamethasone (1 µM, Sigma-Aldrich, D4902) and with an additional 5 mm of sodium DL-lactate solution for metabolic purification of cardiomyocytes (60%, Sigma-Aldrich, L4263). On day 17, purified CMs were refreshed in cardiomyocyte medium consisting of the above-described lactate purification medium with additional 4.5 mm of D(+)-Glucose (Millipore, 1 083 371 000) until day 20. After, cells were dissociated with TrypLE 10× (Thermo Fisher Scientific, A1217702) and cryopreserved in freezing medium comprising 50% Knockout serum replacement (KOSR) (Thermo Fisher Scientific, 10 828 028), 40% cardiomyocyte medium, 10% DMSO (Sigma-Aldrich, D2650), and 0.5% RevitaCell.

### HESC differentiation to cardiac smooth muscle cells

The cardiac dual reporter line NKX2.5^EGFP/+^-ACTN2-^mRubyII/+^ hESC was differentiated to cardiac smooth muscle cells (SMCs) as described.^18^ One day prior the start of the differentiation, hPSC were seeded at a density of 20 × 10^3^ cells per cm^2^ on Matrigel (83 µg protein mL−1)-coated 6-well plates in Essential 8 medium. On differentiation day 0, cardiac mesoderm was induced by the medium with BPEL supplemented with 20–30 ng/mL BMP4 (R&D Systems), 20–30 ng/mL Activin A, (Miltenyi Biotec) and 1.5–2.25 μM CHIR99021 (Axon Medchem). On differentiation day 3 and day 6 the cells were refreshed with BPEL containing 1 µM retinoic acid (Sigma-Aldrich, R2625) and BMP4 (20–30 ng/mL) to induce cardiac specification to the epicardial lineage. On differentiation day 9 pro-epicardial cells were passaged to fibronectin (2 µg/mL) (Sigma-Aldrich, F1141)-coated T175 flasks at a density of 26 × 10^3^ cells per cm^2^ in BPEL medium containing 10 µM TGF-β1 inhibitor SB431542 (Tocris Bioscience, 1614/10). Induction towards cardiac smooth muscle cells was induced by refreshing the medium to BPEL with TGF-β1 (PeproTech, 100–21) (5 ng/mL) and bFGF-2 (Miltenyi Biotec, 130-093-564) (10 ng/mL) on differentiation day 10 and day 12. Afterwards, cells were maintained in full SmGM-2 medium (Lonza, CC-3182) until day 20, when they were cryopreserved at passage number 1 in CryoStor CS10 (StemCell Technologies, 0 7930).

### HiPSC differentiation to endothelial cells

LUMC0054iCTRL#2 hiPSCs were differentiated to endothelial cells as previously described.^18^ Briefly, mesoderm induction was initiated at differentiation day 0 by refreshing with BPEL medium supplemented with CHIR99021 (8 µM). On days 3, 6, and 9, cells were refreshed with vascular specification medium consisting of VEGF (50 ng/mL) (Miltenyi Biotec, 130-109-384) and SB431542 (10 µM) in BPEL. HiPSC-endothelial cells were isolated on day 10 using CD31-Dynabeads (Thermo Fisher Scientific), as previously described.^19^ Afterwards, isolated hiPSC-endothelial cells were expanded for 3–4 days in Human Endothelial-serum-free medium (EC-SFM) (Thermo Fisher Scientific, 11 111 044) supplemented with 1% human platelet-poor serum (Biomedical Technologies, BT-214), VEGF (30 ng/mL) and bFGF-2 (20 ng/mL) (Miltenyi Biotec, 130-093-564) and cryopreserved at passage number 1 in CryoStor CS10.

### Cardiac fibroblast expansion

Human adult cardiac fibroblasts (FBs) were purchased from Promocell (C-12375) and expanded according to their protocol.^20^ Briefly, a T175 cell culture flask (Greiner) was incubated (at 37°C and 5% CO2) with 12 mL of FGM-3 (Promocell, C-23130) for 30 min. After thawing at 37°C, the cells were transferred from the cryovial to a cell culture flask containing the FGM-3 with an additional 18 mL of FGM-3. Whenever the cells reached a 70–90% confluency in the flask, the cells were passaged; this process was repeated until reaching 11 passages. Then, FBs were frozen at a final concentration of 150 × 10^3^ cells 0.5 mL^−1^ in freezing medium made from 50% KOSR (Thermo Fisher Scientific, 10 828 028), 40% FGM-3, 10% DMSO (Sigma-Aldrich, D2650), and 0.5% RevitaCell.^9^

### µ-EHTs fabrication and formation

The µ-EHTs were prepared and seeded according to the protocol as previously described.^18^ Polydimethylsiloxane (PDMS) (Sylgard 184 Silicone elastomer kit, Dow Corning, USA) was cast onto the positive mould and cured in an oven at 65°C for 4–24 h. After curing, the PDMS was removed from the mould, in- and outlets were punched using a 1 mm biopsy punch (Robbins Instruments, USA), and the individual chips were cut out. Each chip was exposed to air plasma (50 W) for 40 s (Cute, Femto Science, South Korea) and bonded onto a glass PDMS spin-coated glass slide. Before seeding, the chips were coated with 1% Pluronic F-127 (P2443-250G, Sigma-Aldrich) in phosphate-buffered saline (PBS) for 20 min at room temperature (RT) after which the solution was aspirated.

3–4 days prior to chip seeding, endothelial cells (ECs) were thawed and cultured on 0.1% gelatin-coated plates in EC-SFM supplemented with 1% human platelet-poor serum, VEGF (30 ng/mL) and bFGF-2 (20 ng/mL) and refreshed two days after thawing. Prior to seeding, the cells were dissociated with TrypLE 1× (Thermo Fisher Scientific, 12 563 029) for 3 min at 37°C, 5% CO2, centrifuged for 3 min at 300 g, resuspended in CM-TDI medium supplemented with VEGF (50 ng/mL) and bFGF-2 (5 ng/mL) and counted. CMs, FBs, and SMCs were thawed, centrifuged at 240 g for 3 min, then resuspended in CM-TDI medium supplemented with bFGF-2 (5 ng/mL) and VEGF (50 ng/mL), and then counted. The CMs, ECs, FBs, and SMCs were then mixed in a ratio of 60:25:10:5, respectively, to obtain a final concentration of 13.2 × 10^3^ cells/µL (59 × 10^3^ cells per tissue)

Per chip 60 µL of gel-cell mixture was made by mixing 10% of Fibrinogen (2 mg/mL final concentration, Sigma-Aldrich F8630), 10% Matrigel (1 mg/mL final concentration), 1% aprotinin (final concentration 2.5 µg/mL, Sigma-Aldrich, A1153), and 80% of cells. Right before seeding, 0.3% thrombin (Sigma-Aldrich, T7513) was added to the gel-cell mixture. Immediately after adding the thrombin, the mixture was mixed and seeded into the chip by pipetting it into the top channel. Subsequently, the mixture was removed from the top channel while the top resistance ensured that the gel-cell mixture remained in the chambers. The gel was polymerized in the chambers for 10 min at RT. The appropriate culture medium was added by placing two pipettes with 45 µL in one side and two with 190 µL on the other side. µ-EHTs were maintained at 37°C and 5% CO2. 24 h after seeding, medium was refreshed, supplemented with DAPT (StemCell Technologies, #72 082) (10 µm), for 24 h. µ-EHTs were maintained at 37°C and 5% CO2 in a rocker platform and refreshed every two days.

### Contraction force measurements

Between day 8–10 after tissue formation, contraction force was measured as initial timepoint and again after 24 h. The tissues were stimulated at 1.5 Hz (10 ms, 50 mV) and video recordings of 5 s per tissue were made. Analysis was done using the software as described in.^21^

### Drug administration and tracer uptake

Between days 8 and 10 post-tissue formation, 48 h prior to tracer injection, Doxorubicin (D1515-10MG, Sigma-Aldrich) was administered at a concentration of 1 µM^5^ and incubated for 24 h at 37°C with 5% CO₂. Control samples, referred to as Control (DOXO), were treated with the vehicle (DMSO) only. After 24 h the complete medium was refreshed.

To inhibit P-gp function, µ-EHTs were treated with Tariquidar (TRQ) for 30 min before the [^18^F]MC225 incubation. Tariquidar (Bio-Techne Ltd., Abingdon, UK) was dissolved in a solution of water (60%), PEG400 (25%, Sigma-Aldrich), TWEEN 20 (10%, Sigma-Aldrich), and DMSO (5%, Sigma-Aldrich),^22^ to a final concentration of 200 nM, as previously described.^23^ Control samples, designated as the control (TRQ) group, received only the TRQ vehicle.

Directly after the pretreatment, the tissues were washed with PBS and then underwent a subsequent incubation with [^18^F]MC225 at a concentration of 1 MBq/mL for 30 minutes.^24^ The production and quality control of [^18^F]MC225 were conducted according to previously established protocols.^5,25^ Post-incubation, the radioactive solution was removed, and the chips were washed 3 times with PBS to eliminate unbound radiolabel.

### Micro-PET scanning

Following the incubation period, the chips were subjected to scanning using a micro-PET scanner (microPET Focus 220, Siemens Medical Solutions, Malvern, PA, USA), employing a 10 min static scanning sequence. Emission sinograms were adjusted for decay, and transmission scan data were utilized to correct attenuation and scattering. No pre-processing smoothing or filter was applied for the [^18^F]MC225 micro-PET scan. Data from the emission scan were reconstructed into 1 frame (600 s) with a voxel size of 0.9 × 0.9 × 0.8. Regions of interest (ROIs) were designated within the central regions of the tissues and the chip channels. These ROIs were then quantitatively assessed for uptake using specialized imaging analysis software (PMOD, version 4.0, PMOD Technologies Ltd, Zürich, Switzerland). The following formula was used to calculate the relative activity of the heart-on-chip tissues in micro-PET analysis:


$$\begin{array}{rl} \mathrm{Relative}\;\mathrm{uptake}(\mathrm{micro}-\mathrm{PET})\;= & \frac{\mathrm{Radioactivity}\;\mathrm{of}\;\mathrm{tissue}\;\mathrm{chamber}}{\mathrm{Average}\;\mathrm{radioactivity}\;\mathrm{of}\;\mathrm{chip}\;\mathrm{channels}}\\ & \times 100\text{\%} \end{array}$$

### Gamma counter

Following the micro-pet scanning period, tissue samples were extracted from the chip models. Each individual tissue sample was then subjected to quantitatively measure tissue radioactivity by γ-counter (LKB Wallac, Turku, Finland), and values were corrected for decay.


$$\begin{array}{rl} \mathrm{Relative}\;\mathrm{uptake}(\gamma -\mathrm{counter})\;= & \frac{\mathrm{Radioactivity}\;\mathrm{of}\;\mathrm{tissue}}{\mathrm{Average}\;\mathrm{radioactivity}\;\mathrm{of}\;\mathrm{control}\;\mathrm{tissues}}\\ & \times 100\text{\%} \end{array}$$

### Autoradiography

In autoradiography, tissues were placed on an activated phosphor storage plate overnight. The storage plates were read using a biomolecular imager (Amersham Typhoon, Global Life Sciences Solutions, USA). Image Analysis Software PerkinElmer (OptiQuant 03.00, Groningen, The Netherlands) was used for detailed visualization and analysis of tracer uptake within the tissues.


$$\mathrm{Relative}\;\mathrm{uptake}(\mathrm{Autoradiography})=\frac{\mathrm{Radioactivity}\;\mathrm{of}\;\mathrm{tissue}}{\mathrm{Average}\;\mathrm{radioactivity}\;\mathrm{of}\;\mathrm{control}\;\;\mathrm{tissues}}\times 100\text{\%}$$

### Tissue fixation and immunofluorescent staining

For 2D-monolayer imaging, CMs (3 × 10^4^ cells), ECs (1 × 10^4^ cells), and SMCs (1 × 10^4^ cells) were seeded on 96-well plates 24 h before fixation with 4% paraformaldehyde (PFA). For tissue fixation, samples were carefully extracted from the chip and subsequently incubated with 4% PFA. Following fixation, tissues underwent a thorough washing process, comprising three washes with PBS to remove excess PFA and preserve sample integrity. Finally, the fixed tissues were stored at 4°C to maintain their structural integrity until further analysis. For the staining, the tissues were washed at RT with PBS, blocked for non-specific binding with 3% BSA (Sigma-Aldrich, A9418), 0.3% Triton-X 100 (Sigma-Aldrich, T8787)), and 0.1% Tween20 (Merck, P9416) in PBS overnight at 4°C. Primary antibodies were diluted and added in antibody buffer, targeting P-glycoprotein (1:100, Thermo Fisher Scientific, MA1-26528), VE-cadherin (1:100, Biotechne, AF938), cTnT (1:250, ProteinTech, 15513-1-AP), and α-actinin (1:800, Sigma-Aldrich, A7811) and then incubated for 48 h at 4°C. Then, tissues were washed at RT with 0.3% Triton-X 100 (3 × 20 min), and secondary antibodies (1:500, AlexaFluor, Fisher) were added overnight at 4°C and protected from light. Next day, tissues were washed three times with PBS for 20 min each at RT with DAPI (1:3000, Thermo Fisher Scientific, D1306) added during the first wash. All incubations were done on a shaker. Finally, µ-EHTs were mounted on a microscope slide with a 0.25 mm spacer (SunJin Lab, IS216) for confocal imaging with a Zeiss LSM 880 microscope.

### P-gp quantification

A macro was written in FIJI (ImageJ)^26^ to automate the quantification of fluorescent signals in microscopy images. For each image, a Gaussian filter with radius 1 was used to reduce background noise and smooth fluorescent signal. Subsequently, Otsu thresholding method^27^ was used to determine the optimal threshold value globally from intensity histogram of the image. Once the binary mask is achieved, measurements are calculated, including mean intensity and total area per image.

### RNA sequencing data

The RNA-sequencing data discussed in this publication are available at the NCBI’s Gene Expression Omnibus (GEO) under GEO accession number GSE232331.

### Statistics

All statistical analyses were performed using GraphPad Prism 9. Descriptive data are presented as mean ± standard deviation (SD), unless otherwise specified. To compare differences between two groups, an unpaired *t*-test was used for data with a Gaussian distribution and equal SD. For data with a Gaussian distribution but unequal SDs, Welch's *t*-test was applied. A *P*-value lower than 0.05 was considered statistically significant.

## Results

### Fabrication and preparation of µ-EHTs for [^18^F]MC225 treatment

**Figure 2:**
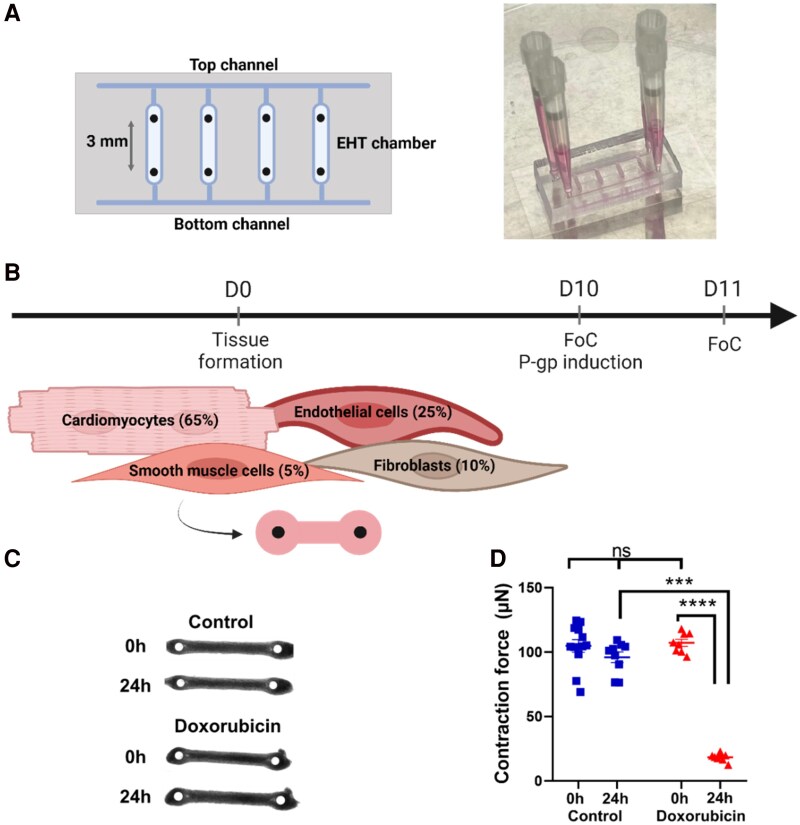
(*A*) Chip design and dimensions (adapted from^18^) and representation of the chip during culture. (*B*) Cell composition of tissue formation and drug administration before contraction force measurements. (*C*) A representative µ-EHT before and after drug administration during contraction force measurements for control and Doxorubicin (*n* = 1 showed). (*D*) Contraction force data (Control *n* = 12 tissues, Doxorubicin *n* = 8 tissues) in which the contraction force reduces significantly after 24 h of a single dose of Doxorubicin. P-values were determined by unpaired *t*-test: ***P < 0.0001, ****P < 0.001.

µ-EHTs were fabricated using the designed chip from^18^ as illustrated in *Figure 2A.* Tissues were seeded on day 0 (D0) using 65% hiPSC-derived CMs, 25% hiPSC-derived ECs, 10% primary FBs, and 5% hiPSC-derived SMCs. After ten days (D10) µ-EHTs were grouped into control and treatment conditions and the contraction force was measured (control tissues at 104.8 ± 4.9 µN and 107.2 ± 2.7 µN for the tissues prior to P-gp induction by Doxorubicin) (*Figure 2B* and *D*). µ-EHTs were treated with 1 µM of DOXO 24 h after drug administration, the contraction force (D11) reduced significantly by 83% (18.3 ± 1.1 µN) compared to the control tissues (8.5% reduction, 95.9 ± 4.1 µN) (*Figure 2D*). After the contraction force measurements of the µ-EHTs on D10 and D11, no morphology differences were observed between the control and Doxorubicin-treated tissues (*Figure 2C*).

### P-gp expression in µ-EHTs increases after doxorubicin treatment

**Figure 3:**
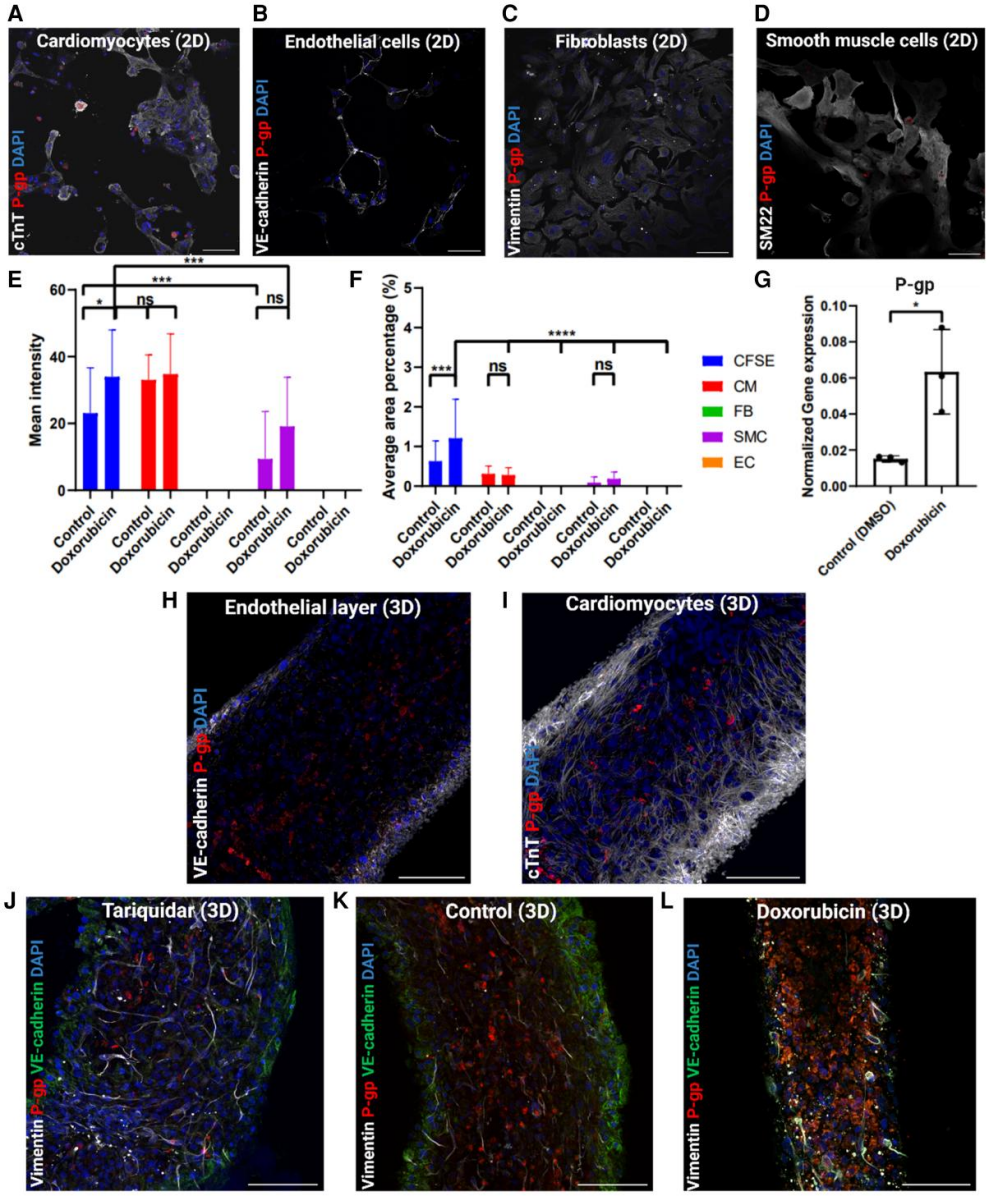
Immunohistochemistry for P-gp expression in µ-EHTs (red, P-gp; blue, nuclei). (*A–D*) P-gp expression in a 96-wells 2D-monolayer culture of (*A*) CMs (white, TroponinT, 3 × 10^4^ cells), (*B*) ECs (white, VE-cadherin, 1 × 10^4^ cells), (*C*) FBs (white, Vimentin, 1 × 10^4^ cells), and (*D*) SMCs (white, SM22, 1 × 10^4^ cells). (*E, F*) Quantification of P-gp expression in co-culture vs. CM, FB, SMC, EC is shown before and after Doxorubicin treatment. In (*E*) significant increase of P-gp expression after Doxorubicin induction is shown in co-culture as well as compared to CMs, FBs, ECs, and SMCs. In (*F*) significant increase of P-gp expression after Doxorubicin induction is shown in co-culture as well as compared to CMs, FBs, ECs, and SMCs. In (*G*) previously generated RNA sequencing data shows that Doxorubicin significantly increases P-gp gene expression after 24 h. Data are presented as the mean ± standard deviation, with P-values determined by unpaired *t*-test: ****P < 0.0001, ***P < 0.001, and *P < 0.05 (*H, I*) A 3D- µ-EHT (*n* = 1 tissue) in which the endothelial layer (*H*, white, VE-cadherin) is shown with P-gp expression inside the tissue, vs. the P-gp expression inside the CMs (*I*, white, troponin T). (*J–L*) P-gp expression in Tariquidar (*J*, *n* = 1 tissue) treated µ-EHT is not different compared to control (*K*, *n* = 1 tissue) µ-EHTs. Increased P-gp expression in µ-EHTs after drug-induced cardiotoxicity by Doxorubicin (*L*, *n* = 1 tissue) treatment. (white, Vimentin; green, VE-cadherin) Scale bar = 100 µm.

After the µ-EHTs were treated with the P-gp inducer Doxorubicin and inhibitor Tariquidar, the tissues were fixated for immunofluorescent staining. Notably, we observed P-gp expression in the hiPSC-derived CMs and SMCs (see Supplementary material online, *Figure S3A* and *D*, respectively), but not in the ECs or FBs (see Supplementary material online, *Figure S3B* and *C*, respectively) that were cultured as a 2D-monolayer. After Doxorubicin induction, there was an increase of P-gp expression in the CMs and SMCs (*Figure 3A* and *D*, respectively), and no expression in the ECs or FBs (*Figure 3B* and *C*, respectively). Notably, ECs continued to show no P-gp expression ever after induction. After co-culture (CFSE) of all cell types as 2D-monolayer, P-gp was expressed in the CMs and SMCs before and after Doxorubicin induction (see Supplementary material online, *Figure S3E* and *F*, respectively). P-gp expression was quantified by creating a binary mask (see Supplementary material online, *Figure S3G* and *S3H*), and a significant increase was observed in intensity after Doxorubicin induction in the co-culture, but not in the CMs, SMC, FB, and ECs individually. In the co-culture there was a significant increase in intensity compared to the ECs, FBs, and SMCs, but not compared to the CMs. The average area of P-gp expression also significantly increased after Doxorubicin induction in the co-culture (*Figure 3F*). Compared to the CMs, ECs, FBs, and SMCs, there was a significant difference after Doxorubicin induction with the co-culture. There was no significant difference after Doxorubicin induction for the CMs, SMC, FB, and ECs individually. To further assess the P-gp expression at the molecular level, previously generated data by RNA-sequencing was used in which EHTs made from only CMS were cultured.^5^ In *Figure 3G* it is shown that the gene expression significantly increased after 24 h of Doxorubicin compared to DMSO (control) in CMs.

After tissue formation and culturing the µ-EHTs, we observed that P-gp expression was localized particularly in the hiPSC-derived CMs inside the tissues as shown in *Figure 3H* and *I*. As previously described by *C. Cofiño-Fabres et al.*, the ECs self-organize as a layer around the tissue and all other implemented cell-types organize randomly by cell-cell interactions. However, no P-gp was expressed in the ECs (*Figure 3H*) in the µ-EHTs as it occurs *in vivo,* but P-gp expression was specifically observed inside of the CMs (*Figure 3I*). As expected, Tariquidar-treated tissues did not show a difference in expression levels of P-gp compared to the control tissues (*Figure 3J*). This is consistent with previous studies, which show that Tariquidar does not influence the expression of P-gp but only inhibits its function^24^ After P-gp induction, the expression of P-gp was expected to increase significantly in the cells as described before^6^ After Doxorubicin treatment, we saw that there was upregulated P-gp expression in the cardiomyocytes of Doxorubicin-treated tissues (*Figure 3L*) in comparison to the control tissues (*Figure 3K*), which indicates that P-gp was successfully induced.

### [^18^F]MC225 to evaluate the function of P-gp in µ-EHTs

**Figure 4:**
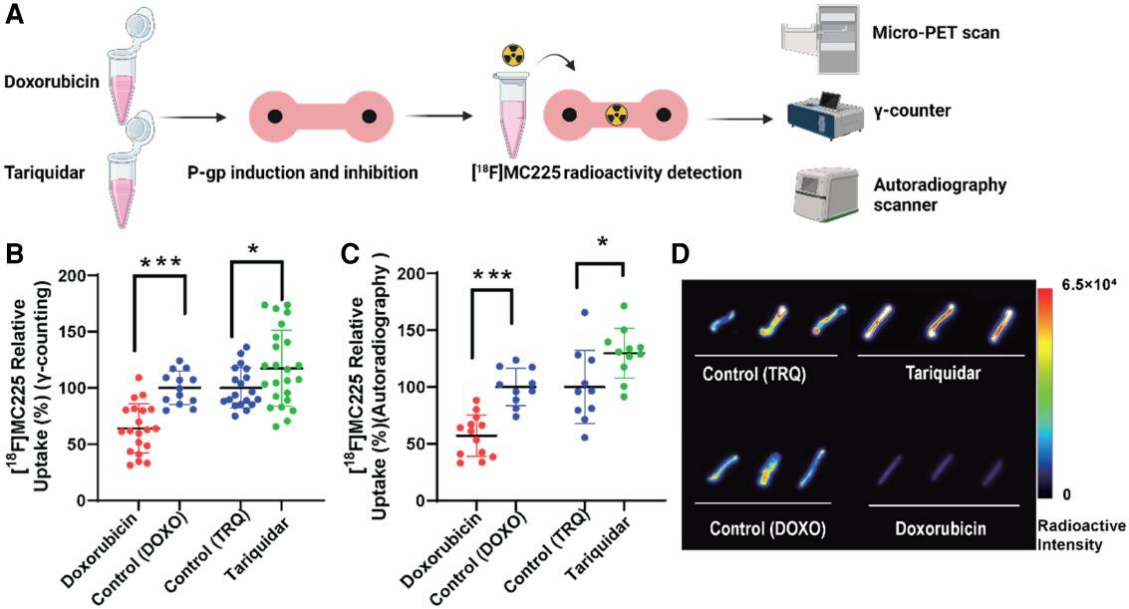
(*A*) Workflow for incubating and radioactivity detection (*B*) Relative uptake as percentages for the Tariquidar (*n* = 19 tissues), Doxorubicin (*n* = 20 tissues), and control groups (*n* = 24 tissues and *n* = 13 tissues, respectively) detected by γ-counters. (*C*) The relative uptake percentages for the Tariquidar (*n* = 11 tissues), Doxorubicin (*n* = 13 tissues), and control groups (*n* = 10 tissues and *n* = 10 tissues, respectively) were determined through autoradiography detection. (*D*) Display of radioactive imaging following treatment with Tariquidar, Doxorubicin, and control groups. Data are presented as the mean ± standard deviation, with P-values determined by unpaired *t*-test: ***P < 0.001, and *P < 0.05.

To assess P-gp function in µ-EHTs, the radioactive tracer [^18^F]MC225 designed specifically to P-gp function was administered to the tissues. As shown in *Figure 4A*, tissues were incubated separately with a P-gp inhibitor (Tariquidar) and inducer (Doxorubicin), followed by incubation with the [^18^F]MC225 tracer. After incubation, tissue radioactivity was measured using a γ-counter, micro-PET, and autoradiography scanner.

The maximum Intensity Projection (MIP) PET imaging was conducted using the micro-PET system to visualize the entire chip in both the Tariquidar-treated tissues and the controls (see Supplementary material online, *Figure S1A*). The imaging effectively visualized the complete platform including the channels and tissues. However, distinguishing the chip channels from the tissues remained challenging. Notably, higher radioactivity was observed accumulating at the injection site. To quantify the tissues, the ratio of tissue radioactivity to background was calculated, as shown in Supplementary material online, *Figure S1B*. Following Tariquidar exposure, this ratio increased from 0.86 ± 0.14 (*n* = 22) to 0.91 ± 0.18 (*n* = 25) (see Supplementary material online, *Figure S1C*).

After micro-PET imaging, the tissues were collected for γ-counter analysis. To facilitate comparison between Doxorubicin and Tariquidar, the uptake in these groups was normalized with the uptake of their respective control groups to the relative uptake in percentages. As shown in *Figure 4B*, compared to the control group of Tariquidar (Control (TRQ), 100%, *n* = 19), the Tariquidar group exhibited a higher uptake (117.5 ± 33.67%, *n* = 24) (*P* = 0.035), while the Doxorubicin group exhibited a lower uptake (63.97 ± 21.89%, *n* = 20) (*P* < 0.001) compared to the control group of Doxorubicin (Control (DOXO), 100%, *n* = 13). This indicates that [^18^F]MC225 can detect both the inhibition and induction of cardiac P-gp function with the γ-counter.

The autoradiographs illustrate tissue imaging, which indicates a consistent and effective uptake of [^18^F]MC225. In terms of relative uptake, compared to the control group of Tariquidar (Control (TRQ)) (100%, *n* = 10), the Tariquidar group showed a higher relative uptake (129.6 ± 21.82%, *n* = 11, *P* = 0.022), while the Doxorubicin group had a lower relative uptake (57.06 ± 18.18%, *n* = 13, *P* < 0.001) compared to (Control (DOXO), 100%, *n* = 10) (*Figure 4C*). *Figure 4D* depicts radioactive imaging following treatment with Tariquidar, Doxorubicin, and its control. The radioactivity in the Tariquidar group was higher than that of the control (Control (TRQ)), while the radioactivity in the Doxorubicin group was lower than that of its control. This indicates that [^18^F]MC225 can detect both the inhibition and induction of P-gp function with autoradiography.

## Discussion

In this study, we demonstrated that it was feasible to measure cardiac P-gp function in the µ-EHT platform. Moreover, we validated the measurement of P-gp function with [^18^F]MC225 under three conditions: normal (control), inhibition (Tariquidar) and induced (Doxorubicin). Compared to the control group, the Tariquidar-treated group exhibited a 17.5% increase in tracer uptake. Conversely, the group treated with Doxorubicin showed a 36.3% decrease in uptake. 24 h after P-gp induction, the CMs’ contractile function was disrupted and significantly reduced temporarily. The uptake of Doxorubicin in the CMs induced upregulation of P-gp expression, resulting in less uptake of [^18^F]MC225. The ability of the µ-EHTs to recover their contraction force over time, as previously described in,^5^ indicates that P-gp function was successfully induced while maintaining cardiomyocyte viability. These results confirm the effectiveness of [^18^F]MC225 in monitoring cardiac P-gp activity in the µ-EHTs. This study sets the stage for future research using P-gp activity to assess the efficacy and safety of novel cardiovascular drugs in 3D-*in vitro* models such as our heart-on-chip model for µ-EHTs.

P-gp functionality can be non-invasively and effectively assessed by PET imaging. Previous studies have demonstrated the efficacy of numerous PET tracers, including [^11^C]Verapamil and [^18^F]MC225, in effectively evaluating P-gp function at the human blood-brain barrier (BBB).^8^ However, as [^18^F]MC225 was developed to track both upregulation and inhibition, this P-gp tracer can measure possible drug-induced cardiotoxic effects more accurately.^9,28^ In this study, we initially evaluated [^18^F]MC225 to measure cardiac P-gp function using our established heart-on-chip model. [^18^F]MC225 exhibited significantly different uptake levels in tissues treated with the P-gp inhibitor Tariquidar and the P-gp inducer Doxorubicin, compared to their control groups. Furthermore, we observed that P-gp was expressed in the CMs which increased after induction. Currently, there are limited studies on cardiac P-gp, but changes in expression are linked to various cardiac diseases or drug-induced cardiotoxicity.^9^ These findings suggest that [^18^F]MC225 can detect changes of cardiac P-gp function in 3D-µ-EHTs. Therefore, the combination of [^18^F]MC225 and 3D-µ-EHTs offers significant potential to facilitate a more precise determination of potential drug-induced cardiotoxicity during drug development of novel compounds, in which optimal dosages could be tailored to individual cardiac P-gp activity levels. The use of 3D *in vitro* models and application of hiPSC-derived cells could facilitate specific disease lines to develop personalized medicine and minimize the risk of adverse effects associated with drug-induced cardiotoxicity.

Both the US Food and Drug Administration (FDA) and the European Medicines Agency (EMA) require evaluation of drug candidates for P-gp interaction potential, including transport, inhibition, and related drug-drug interactions.^29^ Widely utilized for this purpose are cell lines like Caco-2 (human-derived immortalized adenocarcinoma) and MDCK-MDR1 (canine-derived derived cell-line transfected with human P-gp).^30,31^ These are valuable tools for assessing the P-gp substrate activity of pharmaceuticals and drug candidates. Nevertheless, these models do not address cardiac-specific interactions due to lack of morphologic and structural representative models. In our current research, the employment of 3D *in vitro* models and µ-EHTs to assess cardiac P-gp functionality could have future impact for drug development and cardiotoxic research. Compared to traditional cell lines and 2D models, this innovative platform utilizes hiPSCs to differentiate into specific cardiac subtypes, such as CMs, SMCs and ECs. This approach provides a more complex and mature cellular architecture, enabling a more accurate simulation of physiological responses.^13^

Traditionally, animals such as rats and mice were used in the evaluation of novel PET tracers.^32^ However, it is crucial to consider genetic differences between animal models and humans. The P-gp similarity between rodents and humans is 85% to 87%.^33^ These differences could explain the disparities in animal and clinical studies. In our µ-EHT-platform, a human-like cardiac environment has been established, enabling the simulation of physiological responses. Furthermore, recent advancements in the organ-on-a-chip research field have led to the development of integrated systems that simulate the functions of multiple organs and tissues, often referred to as ‘body-on-a-chip’.^34^ These innovations show the ability to evaluate direct cell-cell interactions between different organs within an *in vitro* model. Consequently, the implementation of organ-on-chip platforms presents advantages for preclinical investigations and development of novel PET tracers. For example, not only tracking cardiac P-gp, but other functionalities such as inflammation, calcification, or myocardial injuries can potentially be studied by using µ-EHTs for the development of novel PET tracers.^35,36^ Therefore, these organ-on-chip models have the potential to diminish reliance on animal models and be translated to clinical total body PET/CT scans.^37–39^

In this study, some limitations remain to be addressed. We evaluated cardiac P-gp function in µ-EHTs with the radiotracer [^18^F]MC225. Our results showed that current PET imaging techniques were unable to distinguish between the tissue and chip channels due to the limited spatial resolution in the µ-EHTs platform. The material of the chips that were used is known to be very absorbative, and the lipophilicity of the tracer could also have played a role in the difficult distinguishment of the channels and tissues. Meer *et al.* found that lipophilic surface coatings can help prevent the absorption of small molecules by PDMS. Such coatings could be considered for future use to mitigate this issue.^40^ Micro-PET offers the advantage of continuously monitoring radioactivity during the tracer's tissue uptake and exclusion processes. This dynamic scanning provides valuable insights into how the tracer interacts with tissues exhibiting varying P-gp functions. Recently, Clement *et al.* experienced the same problems and developed an on-chip PET system capable of achieving a spatial resolution of approximately 0.5 mm,^41^ offering a potential solution to this limitation.

A limitation of the current model is observed in the expression pattern of P-gp. Previous studies have reported that P-gp is highly expressed in endothelial cells.^4^ However, in this model, as shown in the figures, P-gp expression in hiPSC-derived ECs is undetectable. In contrast, hiPSC-derived cardiomyocytes within the µ-engineered heart tissue function effectively. In Doxorubicin-treated models, P-gp expression is notably upregulated, consistent with trends observed in other cell lines and animal studies.^6^ These findings suggest that the µ-EHT model holds potential for studying conditions such as Doxorubicin exposure, as well as ischaemia and reperfusion models,^42^ in which changes in P-gp expression are observed in cardiomyocytes.

## Conclusion

Using [^18^F]MC225, we demonstrated that our µ-EHT platform can be used to measure cardiac P-gp function. This research paves the way for future studies to test the safety and effectiveness of new heart drugs using µ-EHTs. It also highlights the potential of heart-on-chip models to reduce reliance on animal models in the evaluation of new radioactive tracers.

## Lead author biography


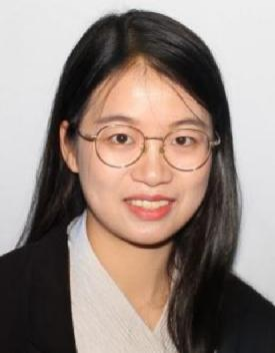


Wanling Liu is a PhD candidate at the University Medical Center Groningen, University of Groningen, The Netherlands. Her research focuses on evaluating cardiac P-glycoprotein (P-gp) function using [18F]MC225 through *in vitro*, preclinical, and clinical studies.


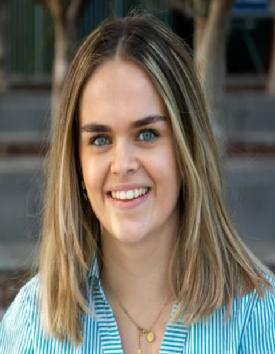


Maureen Dannenberg is a PhD candidate at the University of Twente in Enschede, The Netherlands. She works on heart-on-chip models using IPSC-derived cardiomyocytes and novel engineering techniques to study cardiovascular disease. Her line of work includes using CRISPR-Cas9, engineered heart tissues, and cardiotoxicity screenings. The lead author is appointed as associate professor in clinical radiochemistry at the Department of Nuclear Medicine from UMCG, the Netherlands. Since 2009, he has a position as a clinical radiochemist and is responsible for the PET radiopharmaceutical productions.


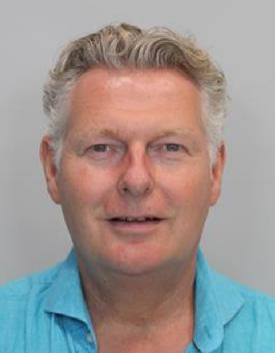


Gert Luurtsema is qualified through translation research and specialized in radiochemistry in relation to PET-tracer development ‘from bench to bed site’ and meets the UMCG requirements for Principal Investigator. His research focused on tracer development to validation of clinical imaging applications. Currently, he supervised > 15 PhD students and is the author of many book chapters and about 150 peer-reviewed scientific articles in high-ranking journals.


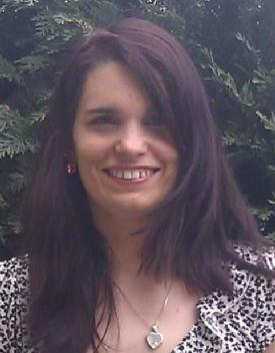


Verena Schwach is an Assistant Professor at the University of Twente, affiliated with the TechMed Centre and the Applied Stem Cell Technologies group of the Department of BioEgineering Technologies. Her research focuses on the development of advanced *in vitro* cardiac models using human pluripotent stem cell-derived cells. Her interdisciplinary approach integrates bioengineering and stem cell biology to create physiologically relevant platforms for cardiac disease modelling, drug screening, and therapy development. She has co-authored numerous peer-reviewed publications in leading journals and actively collaborates across Europe to advance personalized medicine in cardiology.

## Supplementary Material

oeaf150_Supplementary_Data

## Data Availability

Data sharing is not applicable.
