# Supplementary material for: Mapping cardiac drug transport: In vitro assessment of cardiac P-glycoprotein function with [18F]MC225 by using µ-engineered heart tissues
Source: Eur Heart J Open. 2025 Nov 25;5(6):oeaf150. doi: 10.1093/ehjopen/oeaf150 (PMC12662796; doi:10.1093/ehjopen/oeaf150)
Supplement: oeaf150_Supplementary_Data [file oeaf150_supplementary_data.docx]

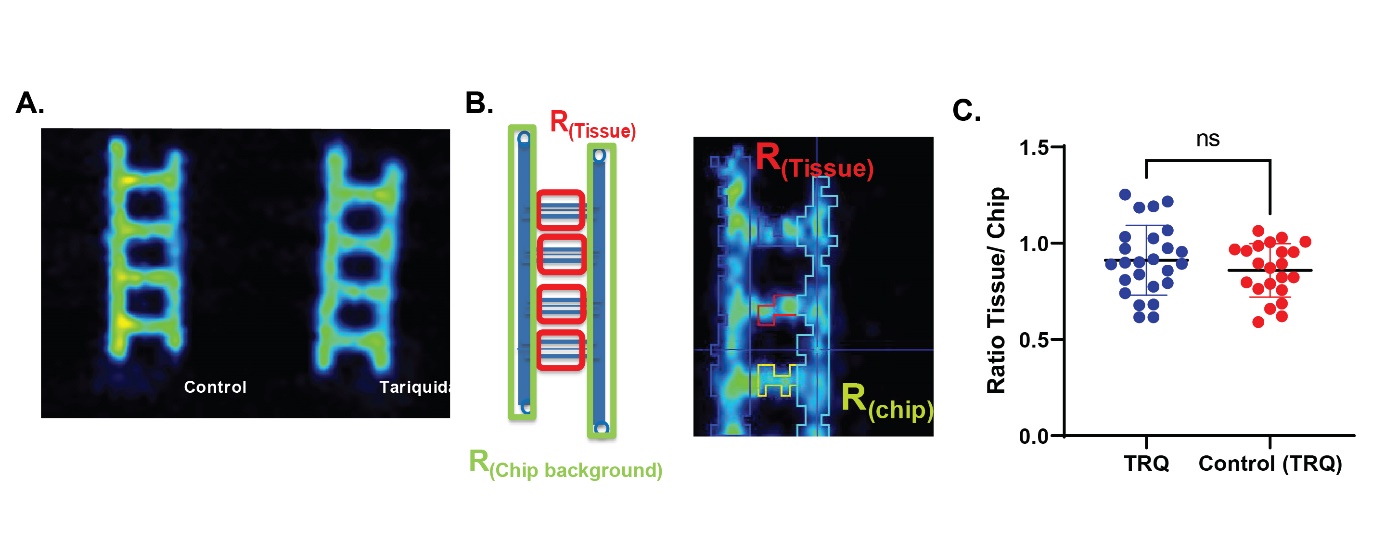


**Supplementary Figure 1: Micro-PET Imaging of µEHTs in the Heart-on-Chip.**

(A) Displays the Maximum Intensity Projection (MIP) PET imaging of the entire Heart-on-Chip using a micro-PET system and compares the Tariquidar-treated group to the control group. The quantification of [^18^F]MC225 uptake is performed using the Volume of Interest (VOI) defined for each tissue section, as shown in (B). (C) Indication that the ratio of tissue radioactivity to background increases after Tariquidar treatment.


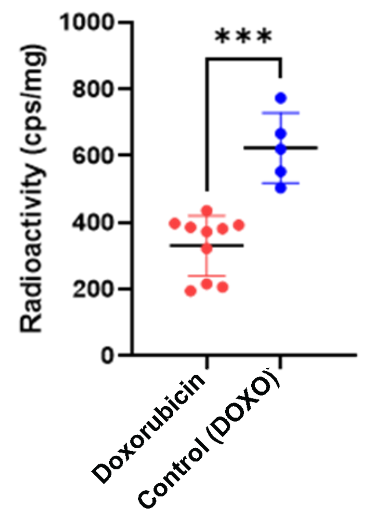


**Supplementary Figure 2: γ-counting analysis of μ-EHTs**

The radioactivity levels in μ-EHTs for both the DOXO and control groups (Control (DOXO)). Data are presented as the mean ± standard deviation, with significance denoted as follows: *** P<0.001


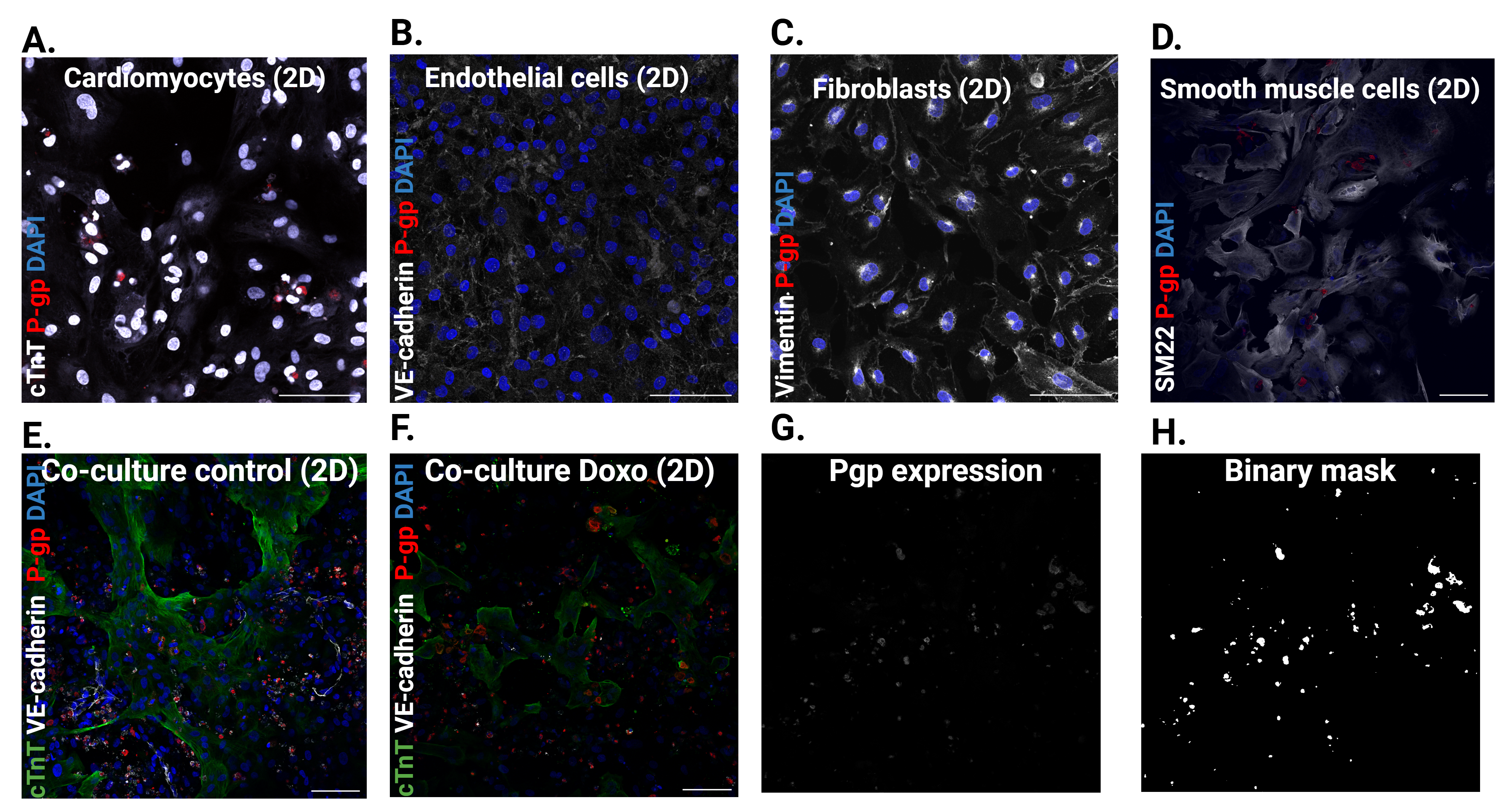


**Supplementary Figure 3: P-glycoprotein expression in different cell-types and quantification of expression.**

Immunohistochemistry for P-gp expression in in a 96-wells 2D-monolayer culture (red, P-gp; blue, nuclei). A, B, C, D) P-gp expression of A) cardiomyocytes (white, cTroponin, 3 x 10^4^ cells) B) endothelial cells (white, VE-cadherin, 1 x 10^4^ cells), C) fibroblasts (white, Vimentin, 1 x 10^4^ cells), D) smooth muscle cells (white, SM22, 1 x 10^4^ cells), E, F) co-culture of CMs (green, cTroponing), ECs (white, VE-cadherin), FBs, SMCs before (E) and after Doxorubicin induction (F). G, H) P-gp expression was quantified by creating a binary mask.
